# A Case Report on Takotsubo Cardiomyopathy

**DOI:** 10.7759/cureus.45285

**Published:** 2023-09-15

**Authors:** Riddhi Chauhan, Bernard Brown, Alam Ahmed, Fadi Yacoub, Sabu John

**Affiliations:** 1 Department of Cardiology, New York City Health and Hospitals/Kings County, Brooklyn, USA

**Keywords:** stress-related cardiomyopathy, ekg abnormalities, transthoracic echocardiogram, cardiomyopathy, takosubo cardiomyopathy

## Abstract

A 71-year-old female with a past medical history of hypertension, seizure disorder, chronic obstructive pulmonary disease, coronary artery disease, chronic kidney disease, open abdominal aortic aneurysm repair complicated by spinal cord infarction resulting in lower extremity paraparesis with chronic urinary retention, and sacral decubitus ulcer initially presented to the emergency department (ED) complaining of a one-week history of chest pain. During her inpatient stay, acute myocardial infarction and pulmonary embolism were ruled out and the patient was hemodynamically stable for discharge until she started experiencing new-onset nausea and dyspnea. Bedside electrocardiogram demonstrated ST elevations in the anterior leads with concomitant T-wave inversions in the inferolateral leads as well as a prolonged QTc. Troponin-HS was elevated at 907.69. Bedside transthoracic echocardiogram (TTE) demonstrated a severely decreased left ventricular ejection fraction of 10%-15% (representing an acute decrease from a left ventricular ejection fraction of 55%-60% from a TTE performed seven days prior). Cardiac catheterization demonstrated mild non-obstructive coronary artery disease and no interventions were conducted. Such signs and symptoms of acute myocardial infarction, without demonstrable coronary artery stenosis, are consistent with stress induced or Takotsubo cardiomyopathy. This phenomenon occurs in approximately 1%-2% of patients presenting with troponin-positive suspected acute coronary syndrome (ACS) or suspected ST-elevation myocardial infarction (STEMI).

## Introduction

Takotsubo or stress cardiomyopathy is characterized by an acute reduction in left ventricular ejection fraction with apical ballooning, a reversible akinesia of the walls of the ventricle, in the absence of an obstructive coronary artery disease. Presenting symptoms can include chest pain, ST-segment elevation and/or T-wave inversions on EKG, or increased troponins. Takotsubo cardiomyopathy was first described in Japan in 1990 in a report of five cases of cardiac arrest patients who were presenting with chest pain and had abnormal EKG findings [1]. The shape of the left ventricle at the end systole resembled the Japanese fisherman's octopus pot - the eponymous Takotsubo [2]. Transient cardiomyopathy affects approximately 1% of patients with troponin-positive acute coronary syndrome (ACS). A typical apical wall motion abnormality is seen in only 60% of patients. Most patients presenting with the syndrome are postmenopausal women who most frequently have symptom onset after an episode of acute physiologic or emotional stress [2].

## Case presentation

A 71-year-old female with a history of hypertension, seizure disorder, chronic obstructive pulmonary disease, coronary artery disease, chronic kidney disease, open abdominal aortic aneurysm (AAA) repair complicated by spinal cord infarction resulting in lower extremity paraparesis with chronic urinary retention, and sacral decubitus ulcer initially presented to the ED complaining of a one-week history of “squeezing chest pain” which “wraps around her back.” The patient also had a two-day history of concomitant shortness of breath. She denied having had any fevers, chills, nausea, vomiting, palpitations, orthopnea, or pain on inspiration. The rest of her review of systems was largely negative.

In the ED, vital signs were: 97.4^o^F, 100 beats/minute, 18 breaths/minute on room air, and 125/66 mmHG. Lab results demonstrated a D-dimer of 2228, a pro-BNP of 310 (<125 pg/mL), and a troponin T of 0.025 (<0.010 ng/mL). The initial chest x-ray was negative for acute findings and a CT chest, abdomen, and pelvis without contrast demonstrated an interval decrease in size of a previously demonstrated aneurysmal sac with an intraluminal aortic endograft extending from the level of the lower thoracic abdominal aorta to the level of the abdominal aortic bifurcation along with a stable aneurysmal dilatation of the iliac vessels. A bedside sonogram in the ED demonstrated an equal right ventricular and left ventricular size; however, parasternal long and short axis views were unavailable due to poor windows. The patient received a full dose of enoxaparin once, morphine for pain control, and was placed on supplemental oxygen in the emergency department. She was admitted for a high suspicion of pulmonary embolism (PE) and possible AAA graft dysfunction. However, repeat bedside echocardiogram did not demonstrate findings consistent with PE, and no acute interventions were performed by vascular surgery as no AAA graft dysfunction was noted. Eventually, the patient was stable for discharge pending placement to a nursing facility. However, later on in the hospital stay, the patient began complaining of new-onset nausea, dyspnea, and anxiety. Vital signs at the time demonstrated a blood pressure ranging in the 160s/110s with a heart rate ranging in the 140s.

**Figure 1:**
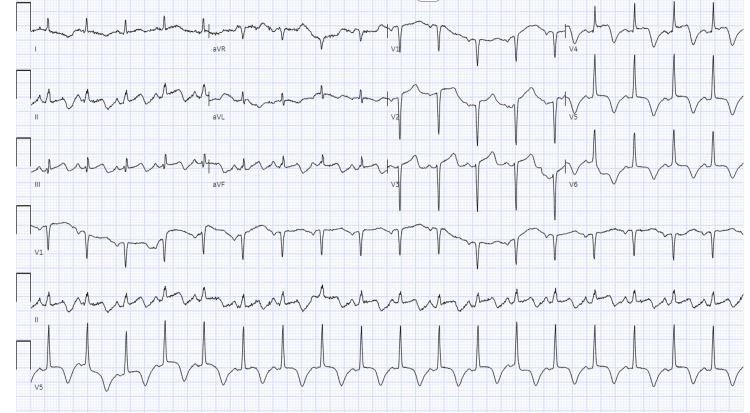
EKG demonstrating ST elevations in the anterior leads (V2-V4) with concomitant T-wave inversions in the inferolateral leads (V5-V6) along with a prolonged QTc.

**Figure 2:**
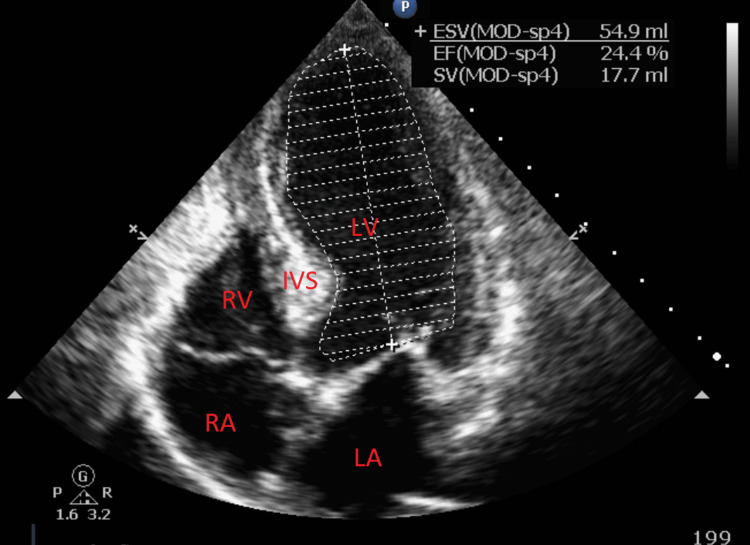
Transthoracic echocardiogram depicting dilated left ventricle with severely reduced ejection fraction. LV-Left Ventricle, LA-Left Atrium, RV-Right Ventricle, RA-Right Atrium, IVS-Intraventricular Septum

EKG at the time demonstrated ST elevations in the anterior leads with concomitant T-wave inversions in the inferolateral leads along with a prolonged QTc (Figure 1). A transthoracic echocardiogram (TTE) performed at the bedside demonstrated a severely decreased left ventricular ejection fraction approximated to be 20%-25%. There was also notable apical, septal, lateral, and anterior wall akinesis along with a hypokinetic inferior wall (Figure 2). This indicated a possible acute left anterior descending artery occlusion with a wraparound left anterior descending artery supplying the apical and distal inferior wall. The right ventricular function was noted to be within normal limits. These TTE findings represented an acute change from the normal TTE findings from seven days prior which demonstrated an ejection fraction of 55%-60% and normal ventricular function.

**Figure 3:**
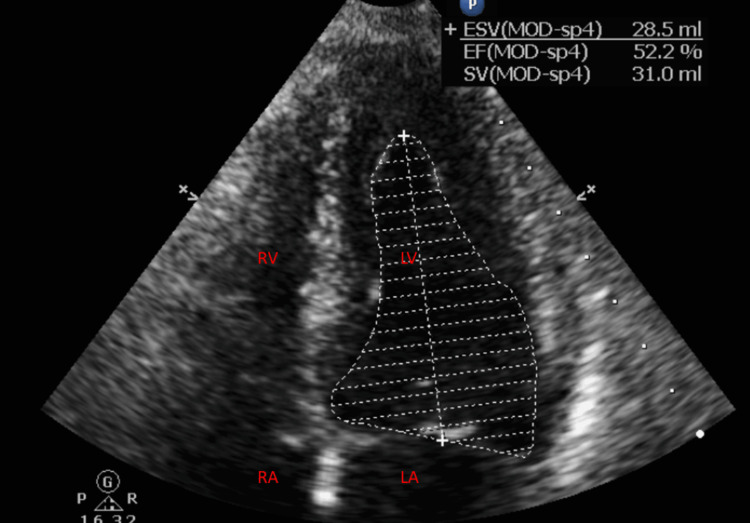
Transthoracic echocardiogram depicting the heart of the patient returning to normal size and with normal ejection fraction. LV-Left Ventricle, LA-Left Atrium, RV-Right Ventricle.

**Figure 4:**
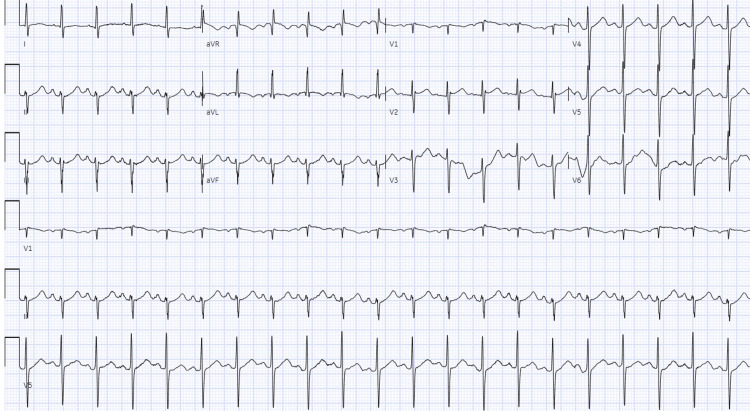
Repeat EKG after left heart catheterization depicting resolution ST and T-wave changes.

A code heart was called; the patient received 324 milligrams of aspirin, 300 milligrams of clopidogrel, and a bolus of heparin. Troponin-HS at that time was elevated at 907.69. A repeat troponin-HS was 879. The patient was transferred to another facility for a left heart catheterization which demonstrated mild nonobstructive coronary artery disease, tortuous coronary arteries, a first diagonal branch with 50% stenosis, and a distal right coronary artery with 40% stenosis. A repeat TTE seven days later demonstrated a return to normal left ventricular function with an ejection fraction estimated to be up to 50%-55% with resolution of the apical akinesis (Figure 3). Repeat EKG showed resolution of ST elevations and T-wave inversions (Figure 4).

## Discussion

The diagnostic criteria for Takotsubo cardiomyopathy were initially proposed in 2004, which have since been modified to include the following: (1) transient hypokinesis, akinesis, or dyskinesis in the left ventricular mid segments with or without apical involvement; regional wall motion abnormalities that extend beyond a single epicardial vascular distribution; and frequently, but not always, a stressful trigger; (2) the absence of obstructive coronary disease or angiographic evidence of acute plaque rupture; (3) new EKG abnormalities (ST-segment elevation and/or T-wave inversion) or modest elevation in cardiac troponin; and (4) the absence of pheochromocytoma and myocarditis.

The acute phase of Takotsubo cardiomyopathy can cause death due to left heart failure, cardiogenic shock, dynamic intraventricular obstruction with left ventricular intracavitary pressure gradient generation, mitral regurgitation (resulting from chordal tethering as well as systolic anterior motion of the mitral valve apparatus), ventricular arrhythmias, left ventricular mural thrombus formation, and/or left ventricular free wall rupture. These complications should be monitored and treated.

Acute Takotsubo cardiomyopathy is also associated with increased levels of C-reactive protein, leukocytosis, and increased levels of serum catecholamines suggesting that catecholamines may be involved in a systemic inflammatory response [1]. Several studies have also reported that cardiac biomarkers generally peak when drawn at the time of initial acute presentation. However, they do not appear to follow the slow rise and fall kinetics observed with conventional acute coronary syndromes or myocardial infarction. Cases of either spontaneous or provocable multivessel epicardial spasms have been present in a few patients, suggesting widespread coronary microvascular dysfunction [2].

The etiology of Takotsubo cardiomyopathy is thought to be multifactorial. For example, emotional stress causes sympathetic hyperexcitability which leads to the massive release of catecholamines. Catecholamines physiologically can enhance myocardial contractions by acting on the myocardial β1/β2 receptors. However, excessive catecholamine release, such as in the case of Takotsubo cardiomyopathy, can cause weakened ventricular wall motion at the apex or increased basal motion. The β1/β2 receptor density is higher in the apical regions as compared to the cardiac basal regions and the sensitivity of the apical adrenergic receptors to catecholamines is also significantly higher than that of the base which in turn leads to the classic apical ballooning seen in Takotsubo cardiomyopathy [1]. In a previous study, it was demonstrated that a high intravenous bolus of epinephrine (but not norepinephrine) produces the characteristic reversible apical depression of myocardial contraction coupled with basal hypercontractility in in-vivo rat models. Functional responses were greater in isolated apical cardiomyocytes compared to basal cardiomyocytes, confirming higher apical sensitivity and response to circulating epinephrine [3,4].

Other physiologic mechanisms that can contribute to the development of Takotsubo cardiomyopathy include direct myocardial toxicity caused by catecholamines; coronary microvascular dysfunction; estrogen deficiency; endothelial dysfunction; and genetic factors. Recently, various malignancies and even COVID-19 have been found to have an association with the onset of Takotsubo cardiomyopathy [1].

Takotsubo cardiomyopathy characteristically presents with transient depressed left ventricular systolic function along with apical and mid-ventricular regional wall-motion abnormalities that generally resolve after a few days to weeks of the initial event. A prior study looking at 22 patients found that all of them achieved baseline cardiac function levels by the time of hospital discharge. At the time of the cardiac event, the left ventricular ejection fraction was estimated to be 29±9% which normalized to an ejection fraction of 63±6% with supportive treatment [5]. After left heart catheterization, adjunctive medical management with beta-blockers, angiotensin-converting enzyme inhibitors in patients without an intracavitary gradient, aspirin, and intravenous diuretics can be used as needed. The prognosis overall is favorable, however, there have been reported cases of death [1].

## Conclusions

Takotsubo cardiomyopathy, also known as “broken heart syndrome” or “stress-induced cardiomyopathy,” continues to challenge clinicians with its diverse clinical manifestations, often mimicking acute coronary syndrome. Left heart catheterization showing non-obstructed vessels is necessary to distinguish Takotsubo from acute coronary syndrome. The prognosis for Takotsubo cardiomyopathy is generally favorable, with most patients recovering left ventricular function within weeks to months. It is possible that her hospitalization due to suspicions of PE or AAA graft dysfunction could have caused extreme stress, leading to sympathetic hyperexcitability, but the exact etiology remains unclear. This case underscores the importance of awareness among healthcare providers about Takotsubo cardiomyopathy, especially in the setting of emotional stress or other triggers. Timely recognition and appropriate management can lead to improved outcomes for affected patients, ensuring that they receive the best possible care during their recovery.
